# Assessing Differential Binding of Aggregation-Induced Emission-Based Luminogens to Host Interacting Surface Proteins of SARS-CoV-2 and Influenza Virus–An *in silico* Approach

**DOI:** 10.3389/fmicb.2021.766351

**Published:** 2021-12-03

**Authors:** Karunakar Tanneeru, Naveen Kumar Bhatraju, Rajesh S. Bhosale, Suresh K. Kalangi

**Affiliations:** ^1^Qstatix Private Limited, Hyderabad, India; ^2^CSIR-IGIB, New Delhi, India; ^3^Department of Chemistry, School of Science, Indrashil University, Rajpur, India; ^4^Amity Stem Cell Institute, Amity Medical School, Amity University Gurugram, Gurgaon, India

**Keywords:** SARS-CoV-2, spike protein, aggregation-induced emission (AIE), rapid diagnostics, H5N1 hemagglutinin protein

## Abstract

Early detection of asymptomatic cases through mass screening is essential to constrain the coronavirus disease 2019 (COVID-19) transmission. However, the existing diagnostic strategies are either resource-intensive, time-consuming, or less sensitive, which limits their use in the development of rapid mass screening strategies. There is a clear pressing need for simple, fast, sensitive, and economical diagnostic strategy for severe acute respiratory syndrome coronavirus 2 (SARS-CoV-2) screening even in resource-limited settings. In the current work, we assessed the *in silico* feasibility of directly labeling virus surface proteins using fluorogenic molecules with aggregation-induced emission (AIE) property. Here, we present the results for binding of two such AIE probes, phosphonic acid derivative of tetraphenyl ethylene (TPE-P) and sulfonic acid derivative of tetraphenyl ethylene (TPE-S), to SARS-CoV-2 spike protein based on *in silico* docking studies. Our results show that both TPE-P and TPE-S bind to angiotensin converting enzyme 2 (ACE2)-binding, and N-terminal domains of SARS-CoV-2 spike protein. Molecular dynamic simulations have revealed specific nature of these interactions. We also show that TPE-P and TPE-S bind to hemagglutinin protein of influenza virus, but the interaction strength was found to be different. This difference in interaction strength may affect the emission spectrum of aforementioned AIE probes. Together, these results form a basis for the development of AIE-based diagnostics for differential detection of SARS-CoV-2 and influenza viruses. We believe that these *in silico* predictions certainly aid in differentially labeling of the both viruses toward the development of rapid detection by AIE probes.

## Graphical Abstract

Proposed design for AIE-based differential detection of SARS-CoV-2 and influenza viruses.

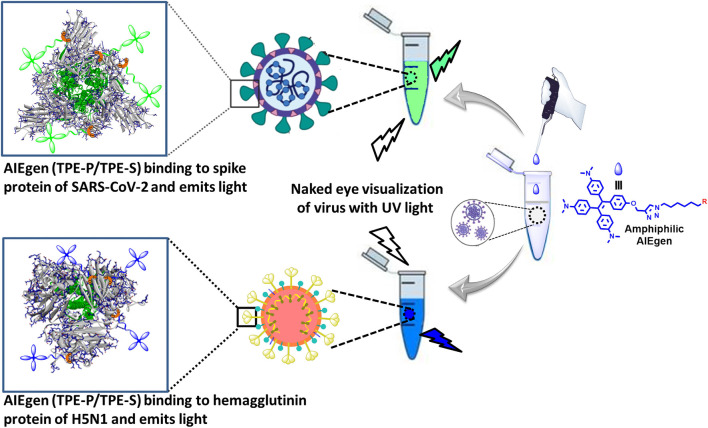

## Highlights

– *In silico* screening and docking exhibited high-affinity binding of phosphonic acid derivative of tetraphenyl ethylene (TPE-P) and sulfonic acid derivative of tetraphenyl ethylene (TPE-S) to angiotensin converting enzyme 2 (ACE2)-binding domain of SARS-CoV-2 spike protein.
– Hydrophilic lysine, arginine, and tyrosine residue interactions are involved in the stability of TPE-P and TPE-S binding to SARS-CoV-2 spike protein.
– *In silico* binding studies exposed possible binding of TPE-S and TPE-P to the N-terminal domain of SARS-CoV-2 spike protein.
– Molecular mechanics Poisson–Boltzmann surface area (MM-PBSA) binding free energy calculation reveals ∼3-fold differential binding strength of TPE-P and TPE-S, toward SARS-CoV-2 and H5N1 hemagglutinin protein, respectively.

## Introduction

Emergence of novel SARS-CoV-2 variants that could evade previously acquired immunity and with relatively higher transmissibility resulted in a devastating COVID-19 second wave in India and worldwide. Although test-trace-treat strategy has helped to mitigate the damage during the first wave, steep rise in the number of daily tests required has soon overwhelmed the diagnostic centers causing increased delays in result reporting during the second wave. Real-time polymerase chain reaction (PCR) is the first-line diagnostic test considered for reliable assessment of SARS-CoV-2 infection (Cheng et al., 2020). However, this test is time consuming and costly and cannot be performed at point-of-care facilities. Alternatively, several immunoassays have been developed that could detect the presence of either antibodies specific to SARS-CoV-2 antigens or specific viral antigens in the patient’s sera (Cheng et al., 2020). Although these tests are fast and easy to perform, low sensitivity and lack of early detection capabilities limit their use in clinical settings. Taken together, shortcomings in the existing SARS-CoV-2 diagnostic pipelines highlight the pressing need for an efficient diagnostic strategy that require no or minimal sample processing and has high sensitivity. Aggregation-induced emission (AIE) is a potential strategy that could fulfill majority of the requirements in this context. AIE techniques offer a potential solution toward developing a fast, sensitive, economical, and easily scalable diagnostic test for SARS-CoV-2 screening with no or minimal sample processing.

Aggregation-induced emission, as the name suggests, utilizes luminogens (AIEgens), a class of materials that are weakly emissive or non-emissive in isolated state, but exhibit enhanced fluorescence in aggregated state (He et al., 2018; Zhao et al., 2020). Apart from overcoming aggregation-caused quenching, a phenomenon that has impeded the performance of traditional fluorophores for long, AIEgens are also loaded with other advantages such as high quantum yield, large Stokes shift, and superior photostability in aggregated state, enabling high-quality fluorescence imaging and long-term fluorescence tracking possible (Zhu et al., 2018; Zhao et al., 2020). Since the first report demonstrating AIE using silole derivatives, AIEgens with a wide range of molecular structures and physiochemical properties have been developed (Mei et al., 2015; Zhu et al., 2018). This has led to a renewed interest into the design of novel bioprobes, with an array of biomedical applications. Of these, targeted biomacromolecule sensing for theranostic purposes is being widely studied (Wang et al., 2018; Zhu et al., 2018; He et al., 2019; Zhao et al., 2020). Recently, several tetraphenyl ethylene (TPE)–based AIEgens have been utilized in the development of theranostic systems for a variety of pathogens including bacteria, fungi, and viruses. Apart from providing ultrasensitivity for diagnosis of the pathogen, such systems could also be used for inactivating the infectious agents (Wang et al., 2018; Zhao et al., 2020). AIE-based systems have also been successfully used for the detection of viruses. At the turn of the last decade, Hatanaka’s group developed novel TPE-based AIEgen probe, α2,6SL-TPE, with 6′-sialyllactosyl oligosaccharide moieties conjugated to TPE core unit, to detect influenza virus (with a limit of detection as low as 105 pfu/100 μL) via AIE fluorescence turn-on mechanism (Kato et al., 2010). Recently, Tang group has reported an AIE-based ultrasensitive virion immunoassay sensing platform (Xiong et al., 2018). In their study, an AIEgen probe TPE-APP, bearing enzymatically cleavable phosphonic acid sites on TPE core, was used to detect EV71 virus even at titers as low as 1.4 copies/μL. Together, these published reports demonstrate the applicability of specially modified AIEgens for specific detection of infectious agents including viruses. Extending on this concept, we made an attempt to investigate the possibility of distinguishing two different viruses based on differential interactions between AIEgen and viral proteins. In the present work, the binding ability of TPE-P and TPE-S to SARS-CoV-2 spike protein and hemagglutinin protein of influenza virus, and the nature of interactions were studied *in silico*.

## Hypothesis

The main idea of this work is to assess the feasibility for the development of AIE-based rapid diagnostic strategy for differential diagnosis of SARS-CoV-2 and influenza. For this purpose, we utilized phosphonic and sulfonic acid derivatives of TPE, a commonly used AIEgen for probing biomolecules. The fluorescence of TPE core could be turned on through restricting the rotary motion of phenyl rings attached to the C=C bond. Several functionalized TPE derivatives for probing different biomolecules are available. Needless to say, the specificity of the interaction is dependent on the functional groups attached. In the present work, TPE-P–bearing phosphonic acid moiety as a head group and TPE-S bearing sulfonic acid moiety as a head group were designed and tested *in silico* for their interaction with coronavirus spike protein (Scheme 1). The reason for using phosphonic acid and sulfonic acid groups to functionalize TPE is based on two facts: (1) phosphonic and sulfonic acid moieties have high affinity toward basic amino acids such as lysine and arginine, and (2) arginine and lysine residues are available on the solvent exposed portions of the spike protein. Further, based on differences in the distribution of the basic amino acid residues between spike protein and influenza hemagglutinin protein, we hypothesized that the binding of AIEgens, TPE-P and TPE-S, to these two proteins is different. Therefore, we assessed binding ability, and nature of interactions, of TPE-P and TPE-S with coronavirus spike protein and hemagglutinin protein *in silico.*

**FIGURE 1:**
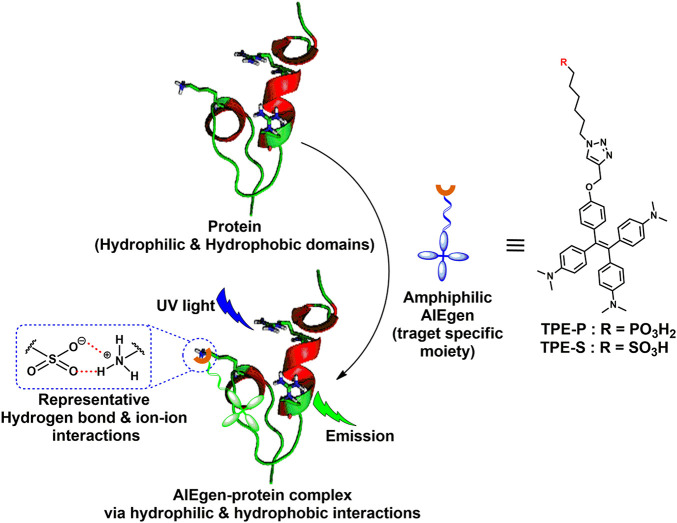
Amphiphilic AIEgen and spike protein complex formation via hydrophilic and hydrophobic interactions; AIEgen-protein complex under UV light irradiation emits light.

## Materials and Methods

### Virtual Screening and Molecular Docking Studies

A 30-molecule database of AIE active derivatives was designed and stored in pdbqt format, and virtual screening was performed against the SARS-CoV-2 spike glycoprotein to understand the most favored binding of the molecules at ACE2-binding site (shown in S1) (Table 1). The screening output were analyzed from the 10 generated conformations. The receptor-binding domain is the ACE2-bound form of SARS-CoV-2 spike glycoprotein crystal structure (PDB_ID: 6M0J, chain A) (Lan et al., 2020). The grid box was generated with the foregoing specifications: 1-Å spacing, box center: *x* = –32.414, *y* = 32.0, *z* = 16.71, and size: *x* = 40, *y* = 44, *z* = 58. Molecular docking studies of the molecules with SARS-CoV-2 spike glycoprotein and H5N1 hemagglutinin were performed using AutoDock Vina program (Morris et al., 2009; Trott and Olson, 2010). Molecular docking of TPE derivative molecules was also performed on N-terminus domain of SARS-CoV-2 spike glycoprotein crystal structure (PDB_ID: 5I08) (Kirchdoerfer et al., 2016). The grid box was generated with the foregoing specifications: 1-Å spacing, box center: *x* = 132.653, *y* = 202.094, *z* = 184.017, and size: *x* = 60, *y* = 48, *z* = 58. Both the TPE-P and TPE-S molecules are docked into one of the antibody-binding sites of H5N1 hemagglutinin protein (PDB_ID: 3S11) (DuBois et al., 2011). The grid box specifications are as follows: 1-Å spacing, box center: *x* = 0.231, *y* = 49.47, *z* = 22.009, and size: *x* = 50, *y* = 74, *z* = 48. For each docking, 20 conformations with top docking scores were generated using default genetic algorithm parameters. In this study, input preparation was carried out using MGLTools 1.5.6, and final docking was performed using AutoDock Vina.

**TABLE 1:**
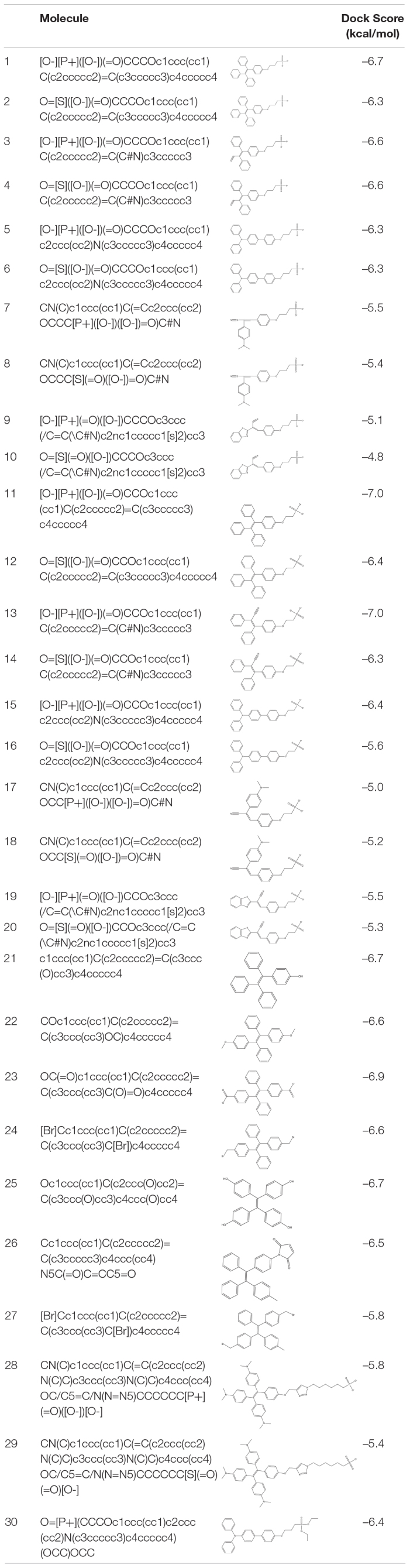
List of virtually screened AIE-active molecules with dock scores against SARS-CoV-2 spike protein.

### Molecular Dynamics Simulations

Molecular dynamics (MD) simulations (100 ns) were performed on TPE-P and TPE-S molecules complexed with SARS-CoV-2 spike glycoprotein receptor–binding domain and H5N1 hemagglutinin. All the MD simulations were performed using Gromacs-2018 version on Ubuntu 18.04 (Van Der Spoel et al., 2005; Hess et al., 2008). The protein force fields were assigned using GROMOS96 53a6 force field (Oostenbrink et al., 2004), and for TPE-P and TPE-S molecules, PRODRG online web server was used to assign the force field (Schuttelkopf and van Aalten, 2004). An edge length with 1.0-nm cubic box used around the protein ligand complexes and SPC water was filled as explicit solvent. The complexes were subjected to steepest descent minimization for initial stabilization and 100 ps of position restrained MD were performed to adjust the water molecules all over the system. A of total 100-ns MD simulations were performed for the whole system using 0.002-ps time step. All the simulations were performed at 300 K constant mean temperature with the V-rescale method thermostat (Bussi et al., 2007), and 1 bar constant pressure was applied using the method of Parrinello and Rahman (1981).

### Molecular Mechanics Poisson–Boltzmann Surface Area Free Energy Calculations

The ligand binding to the protein active site free energy change in terms of Δ*G*_*binding*_ is calculated with the following equation:


$$\Delta G_{binding}=G_{\left(protein+ligand\right)}-\left(G_{protein}+G_{ligand}\right)$$

Where *G_*protein*+*ligand*_* is the total protein–ligand complex free energy, and *G*_*protein*_ and *G*_*ligand*_ are individual free energies calculated in a solvent,


$$G=\left\langle E_{MM}\right\rangle -TS+\left\langle G_{solvation}\right\rangle$$

Where the ⟨*E*_*MM*_⟩ is the average molecular mechanics potential energy in a vacuum. *T* is temperature, and *S* is the entropic contribution, respectively, and the ⟨*G*_*solvation*_⟩ is the free energy of solvation of each component. The free energy (Δ*G*) can be calculated for all individual components for the protein, ligand, and protein–ligand complex.


$$E_{MM}=E_{bonded}+E_{non-bonded}=E_{bonded}+\left(E_{vdW}+E_{elec}\right)$$

Where *E*_*bonded*_ is bonded interaction that included bond, angle, dihedral, and improper interaction energies. The non-bonded interactions (*E_*non*–*bonded*_*) are a combination of both electrostatic (*E*_*elec*_) and van der Waals (*E*_*vdW*_) interaction energies. The energy required to transfer a solute from vacuum into the solvent is the free energy of solvations and is calculated as the combination of *G*_*polar*_ (electrostatic) and *G_*non*–*polar*_* (non-electrostatic) elements.


$$G_{solvation}=G_{polar}+G_{non-polar}$$

The polar solvation free energy is estimated by solving the Poisson–Boltzmann equation, and the non-polar free energy is calculated from the solvent accessible surface area (SASA) non-polar model. In result the free energy of binding was calculated along with its components [van der Waals (vdW), Electrostatic, Polar solvation energy, and SASA energies].

## Results

### *In silico* Docking Shows High-Affinity Binding of Phosphonic Acid Derivative of Tetraphenyl Ethylene and Sulfonic Acid Derivative of Tetraphenyl Ethylene to Angiotensin Converting Enzyme 2-Binding Domain of SARS-CoV-2 Spike Protein

Best binding molecules were chosen based on the virtual screening. The molecules with best binding pose and good score were considered for further molecular docking. From the docking results of the 30 molecules database, TPE derivative and tri-phenyl amine derivates were docked into various locations of the spike protein of the SARS-CoV-2. The ACE2-binding site location was considered for the further analysis. Among the 30 molecules in Table 1, a total of eight molecules (1, 11, 13, 21, 25, 28, 29, and 30) were chosen for further level of docking in AutoDock Vina using 20 generated conformations. From the output data analysis, especially, the molecular docking studies of TPE-P (molecule-28) and TPE-S (molecule-29) molecules were found to have good binding orientation and interaction with ACE2 binding region of coronavirus spike protein (Figure 1 and Supplementary Figure 1). The results of molecular docking were considered primarily based on the orientation of the molecule in the active site and scores. The aromatic TPE region and the six carbon (C_6_) trunk containing phosphonic acid or sulfonic acid head groups were found to interact with the hydrophobic residues and surface polar residues of the spike protein, respectively. The docking conformations were observed to be similar for both TPE-S–spike protein and TPE-P–spike protein with binding affinities of –5.7 and –5.6 kcal/mol, respectively. The TPE moiety was stabilized by the residues Gln498, Asn501, Thr500, and Tyr449, and a strong π–π interaction with Tyr505. The phosphonic acid and sulfonic acid head groups were found to form hydrogen bonds with side-chain NH of Gln409 and the main chain carbonyl oxygen of Asp405. The Lys417 and side chain of Arg408 were also involved in the stabilization of the polar head groups of TPE. The triazole ring stabilized by cation-π interaction with Arg403 and the side chain of Tyr453 and Tyr495 residues.

**FIGURE 1:**
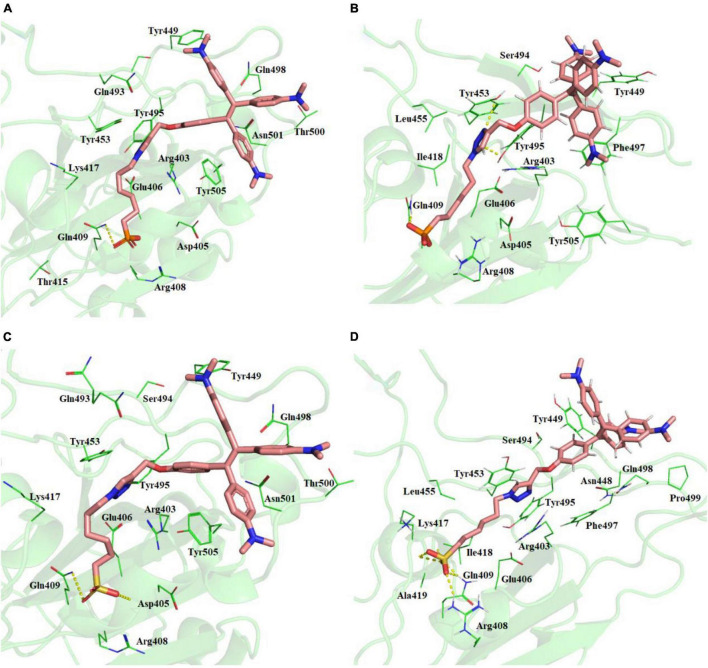
**(A)** Molecular docking of TPE-P into the active site of the ACE2-binding domain of SARS-CoV-2 spike protein. **(B)** The average structure of the TPE-P bound ACE2-binding domain of SARS-CoV-2 spike protein extracted from the last 20 ns of total 100 ns of MD simulations. **(C)** Molecular docking of TPE-S into the active site of the ACE2-binding domain of SARS-CoV-2 spike protein. **(D)** The average structure of the TPE-S bound ACE2-binding domain of SARS-CoV-2 spike protein extracted from the last 20 ns of total 100 ns of MD simulations. The TPE-P and TPE-S molecules are shown in the stick model, and the amino acid residues are shown in line representation. The hydrogen bonds are shown in broken yellow lines.

### Ionic Interactions Involving Lys Residues Are Involved in the Stability of Phosphonic Acid Derivative of Tetraphenyl Ethylene and Sulfonic Acid Derivative of Tetraphenyl Ethylene Binding to SARS-CoV-2 Spike Protein

The MD simulations of the spike glycoprotein with both TPE-S and TPE-P molecules (Figure 1) revealed minor modifications in the binding interactions. During MD simulations, the movement of the TPE-S and TPE-P molecules was observed, especially at C_6_ trunks with polar head region with improved binding. The protein RMSD in complex with both of the TPE analogs was observed at 0.4–0.45 nm, and both the complexes RMSDs were converged after the 30 ns of simulation time. The C_6_ trunk of both polar sulfonic acid and phosphonic acid was moving with high RMSD 0.3–0.35 nm. The phosphate group of the TPE-P formed a hydrogen bond with side-chain NH of Gln409 residue. The TPE-P molecule was stabilized by continuous π–π and CH-π interactions of two phenyl rings of TPE-P with Tyr449. One of the aromatic rings of TPE formed a π–π interaction with Phe497 residue. The triazole ring of TPE-P also formed hydrogen bonds with Tyr453 and Tyr495 residues. The phosphonic acid group was stabilized by Lys417, Tyr421, and Tyr473 residues. One of the aromatic rings of TPE-S molecule formed a π–π interaction with Tyr449. The other aromatic ring of TPE formed a cation-π interaction with the side-chain NH of Asn501 residue. Also, formation of a hydrogen bond between NH of Lys417 residue and the sulfate group of TPE-S was observed during the simulation. The OH group of sulfonic acid was also found to form a trifurcated hydrogen bond with side-chain NH of Gln409, main-chain NH of Arg408, and main-chain NH of Ile418 residues.

### *In silico*–Binding Studies Show Binding of Phosphonic Acid Derivative of Tetraphenyl Ethylene and Sulfonic Acid Derivative of Tetraphenyl Ethylene to Unique N-Terminal Domain of SARS-CoV-2 Spike Protein

**FIGURE 2:**
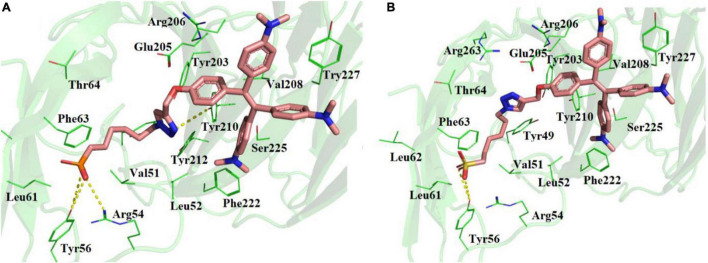
Molecular docking of **(A)** TPE-P and **(B)** TPE-S in the active site of N-terminal domain of SARS-CoV-2 spike protein. The TPE-P and TPE-S molecules are shown in stick model and the amino acid residues are shown in line representation. The hydrogen bonds are shown in broken yellow lines.

Molecular docking studies showed that TPE-P and TPE-S may also bind to the N- terminal domain of SARS-CoV-2 spike protein (Figure 2 and Supplementary Figure 2). The binding site was located near the junction region of N-terminal domain and receptor-binding domain, and the binding affinities were found to be –6.5 and –6.3 kcal/mol, respectively. The binding pocket residues Tyr49, Val51, Lue52, Arg54, Tyr56, Leu51, Leu52, Phe53, and Thr54 surrounded the C_6_ trunk with phosphonic acid and sulfonic acid polar head groups. The Tyr56 formed a hydrogen bond with OH moiety of TPE-S sulfonic acid group. The phosphonic acid polar head formed hydrogen bonds with Tyr56 and Arg54 residues. The TPE moiety of both molecules were stabilized by Glu205, Arg206, Val208, Ser225, and Tyr227 residues. One of the aromatic rings of TPE was observed to form a π–π interaction with the side chain of Tyr227. The side-chain OH of Tyr210 formed a hydrogen bond with the triazole ring of TPE-P molecule. Both the molecules were stabilized by hydrophobic and hydrogen bonds in the active site of the N-terminal domain of SARS-CoV-2 spike protein.

### Differential Binding Patterns of Phosphonic Acid Derivative of Tetraphenyl Ethylene and Sulfonic Acid Derivative of Tetraphenyl Ethylene to SARS-CoV-2 and H5N1 Hemagglutinin Protein

**FIGURE 3:**
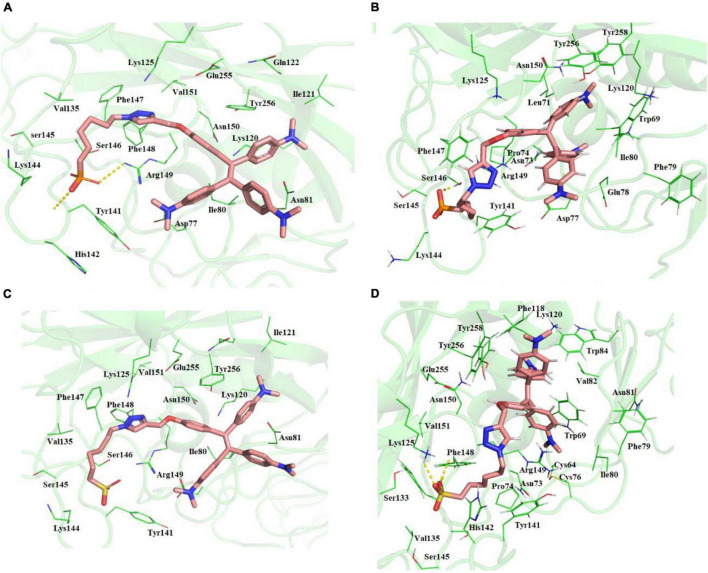
**(A)** Molecular docking of TPE-P into the active site of the H5N1 hemagglutinin protein. **(B)** The average structure of the TPE-P bound H5N1 hemagglutinin protein extracted from the last 20 ns of total 100 ns of MD simulations. **(C)** Molecular docking of TPE-S into the active site of the H5N1 hemagglutinin protein. **(D)** The average structure of the TPE-S bound H5N1 hemagglutinin protein extracted from the last 20 ns of total 100 ns of MD simulations. The TPE-P and TPE-S molecules are shown in the stick model, and the amino acid residues are shown in line representation. The hydrogen bonds are shown in broken yellow lines.

The two molecules TPE-P and TPE-S docked in to the surface site of the H5N1 hemagglutinin protein (Figure 3 and Supplementary Figure 3). Both the molecules were bound to the antibody-binding site comprising the Asp77, Ile80, Asn81, Lys120, Ile121, Gln122, Lys125, Val35, Tyr141, His142, Lys144, Ser145, Ser146, Phe147, Phe148, Arg149, Asn150, Val151, Glu255, and Tyr256 residues. Few of the residues Tyr141, His142, Lys144, Ser145, and Ser146 were reported to be crucial for antibody binding. Molecular docking studies revealed that the TPE-P and TPE-S molecules bind to the hemagglutinin at one of the antibody binding sites with binding affinities of –6.3 and –5.7 kcal/mol, respectively. The TPE region was bound to the hemagglutinin protein with Asp77, Ile80, Asn81, Lys120, Ile121, Gln122, Glu255, and Tyr256 residues. One of the aromatic rings of both molecules formed a π–π interaction with Tyr256 residue. From the molecular docking studies, the polar head sulfonic acid and phosphonic acid showed the presence of non-covalent interactions with Tyr141, Lys144, and Arg149 residues.

Molecular dynamics simulations of the two complexes showed the binding interactions of H5N1 hemagglutinin with TPE-S and TPE-P molecules (Figure 3). The hemagglutinin protein RMSD convergence in complex with the both TPE analogs was observed at 0.6–0.85 nm, and both the complexes RMSDs converged at the end of the 100-ns simulation time. From the trajectory, high fluctuations of the loops were observed, which lead to an increase in the RMSD of the protein. The ligand movement of TPE-P molecule was observed around 0.3 nm, indicating the movement of phosphate C_6_ trunk from its docked position to interact with Ser146 residue, and MD simulation removed the strain of docked conformation, and the TPE-P was free to bind in the antibody-binding site. Although the aromatic rings of the molecules made π–π interaction with Tyr256 residue and stabilized the TPE in the binding site throughout the simulation, a slight movement of the TPE moiety was observed. The TPE-S molecule also showed some fluctuation during the simulation in complex with hemagglutinin. The molecule totally moved from its docked conformation through Arg149 residue support and stabilized in between Arg149 and Tyr141 residues. The phosphate group oxygen atoms formed hydrogen bonds with the main chain elements of Phe147 and Phe148 residues. The side-chain aromatic ring of Tyr141 showed a CH-π interaction with C6 trunk of TPE-S moiety. The C_6_ trunk with sulfonic acid head group was also stabilized by non-bonded interactions with Pro74, and His144 residues.

**FIGURE 4:**
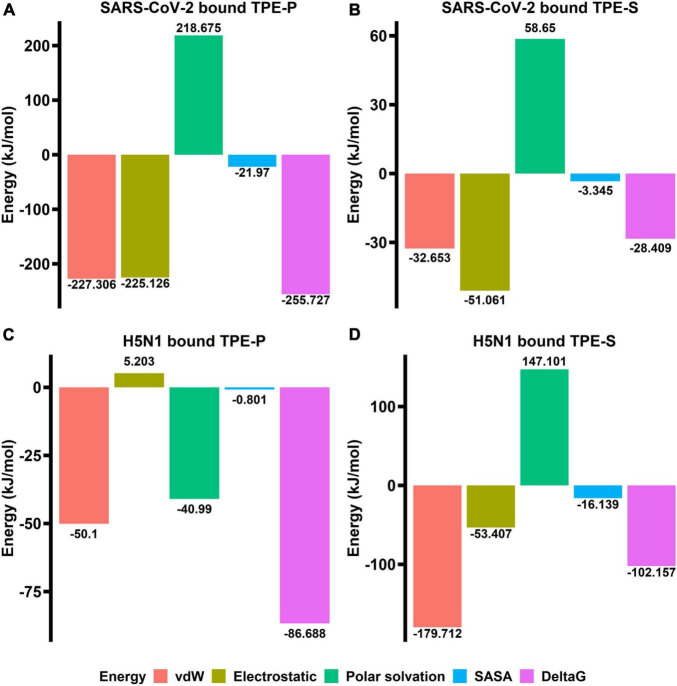
The bar plots **(A–D)** indicating the free energy components (vdW, electrostatic, polar surface, SASA, and ΔG) obtained from MM-PBSA calculations relative to TPE-P and TPE-S bound to SARS-CoV-2 ACE2-binding domain and H5N1 hemagglutinin protein. The energy values are shown in kJ/mol.

The binding free energy revealed that the TPE-P molecule has strong interaction (–255.727 kJ/mol) with the ACE2-binding domain of SARS-CoV-2 spike protein (Figure 4), where the TPE-S has less binding free energy (–28.409 kJ/mol) than TPE-P. The free energy components of TPE-P and TPE-S to SARS-CoV-2 ACE2-binding domain are listed in Table 2. The binding energy values for TPE-P and TPE-S in complex with H5N1 hemagglutinin protein were found to be –86.688 and –102.157 kJ/mol, respectively. The values of free energy components for TPE-P–hemagglutinin and TPE-S–hemagglutinin complexes are listed in Table 2. The MM-PBSA free energy calculation revealed that TPE-P binds more strongly to SARS-CoV-2 spike protein, and TPE-S showed good binding to H5N1 hemagglutinin protein. Together, these results show that the binding of TPE-P and that of TPE-S to hemagglutinin are different and thus may affect the extent by which the molecular motions of the AIEgens are restricted in the bound state. This differential restriction in the molecular motions may, in turn, affect the emission wavelength and/or intensity of the TPE-P and TPE-S molecules.

**TABLE 2:** Table showing values of free energy components between TPE-P, TPE-S, and SARS-CoV-2 and H5N1 proteins.

SARS-CoV-2 RBD–TPE-P complex	
**Free energy component**	**Score (kJ/mol)**
vdW energy	–227.306
Electrostatic energy	225.126
Polar solvation energy	218.675
SASA	–21.97
**SARS-CoV-2 RBD–TPE-S complex**	
vdW energy	–227.306
Electrostatic energy	225.126
Polar solvation energy	218.675
SASA	–21.97
**H5N1 hemagglutinin–TPE-P complex**	
vdW energy	–50.1
Electrostatic energy	5.203
Polar solvation energy	–40.99
SASA	–0.809
**H5N1 hemagglutinin–TPE-S complex**	
vdW energy	–179.712
Electrostatic energy	–53.407
Polar solvation energy	147.101
SASA	–16.139

## Discussion

Rapid screening, together with isolation of infected patients is key to restrict the spread of pandemics such as COVID-19. However, the current reverse transcriptase–PCR–based technique for confirmatory diagnosis of COVID-19 is resource-intensive, time-consuming, and relatively expensive limiting its applicability toward mass screening. Therefore, alternative strategies that could overcome these shortcomings are required. In this context, we propose an AIE-based virus detection system as a potential strategy that could be used for the development of rapid screening methods. The ability of AIE-based techniques for sensitive and rapid detection of viruses has been documented. In order to rapidly develop similar detection strategies for SARS-CoV-2, it is important to identify existing AIEgens that could potentially bind to proteins present on virus surface. The analysis of the three-dimensional structure of SARS-CoV-2 spike protein revealed the presence of basic amino acids, arginine, and lysine, in the ACE2-binding, and N-terminal domains of spike protein. This suggests that negatively charged amphiphilic AIEgens could be able to bind to these domains. Therefore, in order to understand the AIEgen-based direct labeling of SARS-CoV-2, we studied molecular interactions between two variants of a well-known AIEgen, TPE, and different domains of spike protein. The two TPE analogs, TPE-P and TPE-S, were chosen based on virtual screening of 30 molecules listed in Table 1. TPE-P and TPE-S exhibited differences in their binding to SARS-CoV-2 spike protein and H5N1 hemagglutinin protein. TPE-P exhibited ∼3-fold stronger binding to the spike protein compared to hemagglutinin. In contrast, TPE-S exhibited stronger binding to hemagglutinin protein compared to spike protein. In both cases, ∼3-fold difference in the binding strengths was observed. The structure based *in silico* studies revealed the TPE-P and TPE-S molecules interact with both ACE2-binding and N-terminal domains of SARS-CoV-2 spike protein and also H5N1 hemagglutinin protein. Molecular docking and MD simulation studies revealed that the molecules were stabilized by aromatic residue π–π interactions and polar group hydrogen bonding at these sites. The interactions of TPE-P and TPE-S molecules with SARS-CoV-2 spike protein receptor-binding domain open up an ACE2-binding cavity, which can partially accommodate the TPE aromatic moiety. Both polar sulfonic acid and phosphonic acids were stabilized by the Gln409 and Lys417 residues. We expect that the designed AIEgens have advantage of binding either to cleaved monomer or intact trimers with specificity of proposed amino acids toward ACE2-binding domain and N-terminal domain of spike protein, which further amplify the opportunities to increase the sensitivity of the test. The designed TPE-S and TPE-P AIEgens have propeller-shaped three-dimensional aromatic core structure that can undergo low-frequency motions (rotations) in solution and dissipate excitation energy via non-radiative decay process and thus have weak emission. On the other hand, such intramolecular motions are constrained when TPE-S and TPE-P AIEgens are in complex form with SARS-CoV-2 spike protein (in aggregated state) via hydrogen bonding between sticky head group of TPE-S and TPE-P amphiphiles (polar sulfonic acid and phosphonic acids) and NH of Lys417, Gln409, and Arg408 residues. The TPE aromatic core moiety was further stabilized by π–π interaction with Tyr505, which may result in radiative decay upon UV light irradiation. The observed fluctuations of loop regions and molecular movements in the binding site of hemagglutinin protein suggest that the binding affinity of TPE-P is slightly less in comparison to that of SARS-CoV-2 spike protein. SASA and electrostatic energy of bound conformations suggest that the TPE-P molecule binds strongly to SARS-CoV-2 in comparison with H5N1 hemagglutinin. The lack of N-terminal domain in H5N1 hemagglutinin and the TPE-P molecule weak binding in comparison to SARS-CoV-2 spike protein may help differentiating both the proteins in a given sample.

The stabilization of TPE core by π–π and cation–π interactions is necessary for AIE. We have shown that the binding of TPE-P and TPE-S to spike protein involves both π–π and cation–π interactions, which further stabilize the TPE core in the ACE2 receptor–binding domain. The emergence of novel SARS-CoV-2 variants with altered amino acids in the spike protein may cause variations in the binding of TPE derivatives to spike protein. For instance, we have shown that one of the aromatic rings of TPE core forms a cation–π interaction with –NH group of Asn501. However, some of the recent SARS-CoV-2 variants have Tyr in place of Asn at this position (Chen et al., 2021; Dejnirattisai et al., 2021). Such mutations may affect the quality of TPE binding to the spike protein, which may or may not affect the emission patterns. Therefore, the effect of alterations in these amino acids on the AIE patterns of TPE bound to spike proteins forms an important question that requires to be evaluated experimentally. This is, in fact, important for generalization of the AIE-based diagnosis of emerging SARS-CoV-2 variants.

## Conclusion

The present study explores the feasibility of AIE-based differential diagnostic strategy for detection of SARS-CoV-2 and influenza viruses. Our results based on *in silico* virtual screening, molecular docking studies and MD simulations reveal that the designed AIEgens (TPE-P and TPE-S) bind to SARS-CoV-2 spike protein and influenza hemagglutinin protein with different binding strengths. This differential binding may affect the emission spectra and/or intensities of the AIEgens. Although experimental validation is required, these results are encouraging and form the first step toward development of AIE-based differential diagnostics for SARS-CoV-2. The development of such economic, sensitive, and scalable diagnostic strategies with rapid turnover time has tremendous implications in mass screening during epidemics. The only caveat we foresee for these kinds of techniques is the ready availability of high-resolution 3D protein structures.

## Data Availability Statement

The datasets presented in this study can be found in online repositories. The names of the repository/repositories and accession number(s) can be found in the article/Supplementary Material.

## Author Contributions

KT: *in silico* experiments, results analysis, and related write up. NB: literature survey, participating in discussion in streamlining idea, and editing of the article. SK: concept and formulating hypothesis, graphical abstract, and finalization of article. RB: AIE molecule design and related write up. All authors contributed to the article and approved the submitted version.

## Conflict of Interest

KT is employed by the Qstatix Private Limited. The remaining authors declare that the research was conducted in the absence of any commercial or financial relationships that could be construed as a potential conflict of interest.

## Publisher’s Note

All claims expressed in this article are solely those of the authors and do not necessarily represent those of their affiliated organizations, or those of the publisher, the editors and the reviewers. Any product that may be evaluated in this article, or claim that may be made by its manufacturer, is not guaranteed or endorsed by the publisher.
