# Supplementary material for: Assessing Differential Binding of Aggregation-Induced Emission-Based Luminogens to Host Interacting Surface Proteins of SARS-CoV-2 and Influenza Virus–An in silico Approach
Source: Front Microbiol. 2021 Dec 3;12:766351. doi: 10.3389/fmicb.2021.766351 (PMC8682235; doi:10.3389/fmicb.2021.766351)
Supplement: Supplementary file 1 [file Data_Sheet_1.docx]

**Supplementary information**

URunning Title

**Assessing differential binding of aggregation induced emission-based luminogens to host interacting surface proteins of SARS-CoV-2 and influenza virus- an *in silico* approach**

UAuthor information

**Karunakar Tanneeru**P**^1†^**P**, Naveen Kumar Bhatraju**P**^2†^**P**, Rajesh S. Bhosale**P**^3*^** P**and Suresh K. Kalangi**P**^4*^**

1. Qstatix Private Limited, Hyderabad-500035, India. E-mail: karu.tanneeru@gmail.com
2. CSIR-IGIB, Mall road, New Delhi, India 110007. Email: naveenb82@gmail.com
3. Department of Chemistry, School of Science, Indrashil University, Rajpur, Mehsana-382470, Gujarat, India. E-Mail: rajeshbhosale24@gmail.com
4. Amity Stem Cell Institute, Amity University Haryana, Amity Education Valley Pachgaon, Manesar Gurugram (HR)-122413, India.

E-Mail: [30TUskkalangi@ggn.amity.edu](mailto:skkalangi@ggn.amity.edu)U30T †

**Correspondence:**

Dr. Suresh Kumar Kalangi

Amity Stem Cell Institute,

Amity University Haryana,

Amity Education Valley,

Pachgaon, Manesar Gurugram (HR)-122413, India.

E-Mail: [30Tskkalangi@ggn.amity.edu](mailto:skkalangi@ggn.amity.edu)30T

ORCID ID: 0000-0002-7328-9322

† These authors have contributed equally

*These authors have Contributed equally


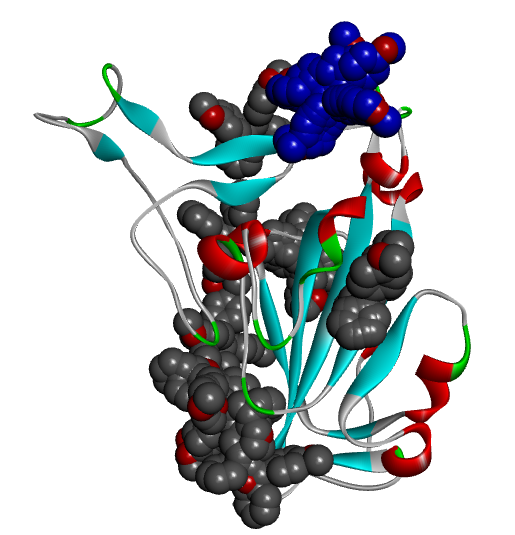


**Figure SI:** Possible binding site on the spike protein of SARS-COV2 identified from the molecular docking using Autodock vina. The blue color molecules located in the ACE2 binding site on the Spike protein.


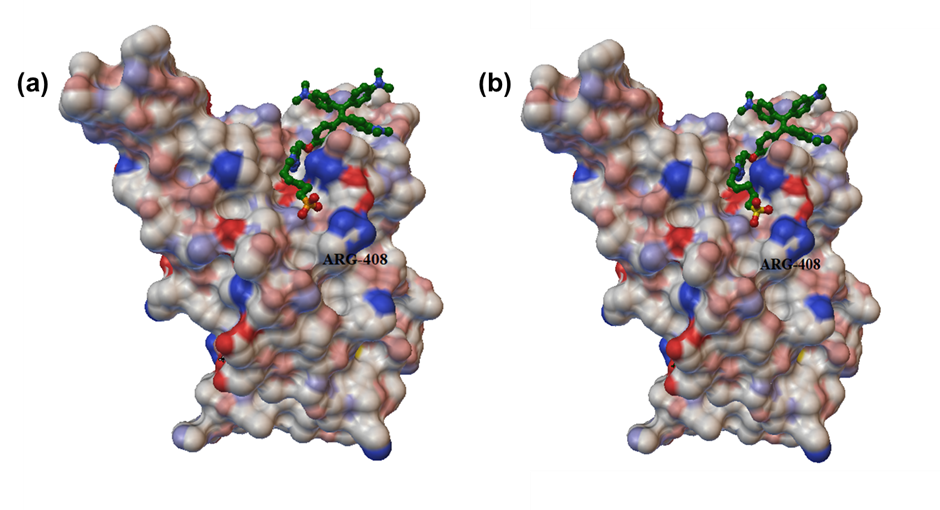


**Figure SII**: Molecular docking of (a) TPE-P & (b) TPE-S in the active site of ACE2 binding domain of SARS-CoV-2 spike protein. The protein is shown in with molecular surface representation. The TPE-P & TPE-S molecules are shown in ball and stick model and the Arg408 amino acid residue location is shown on the protein.


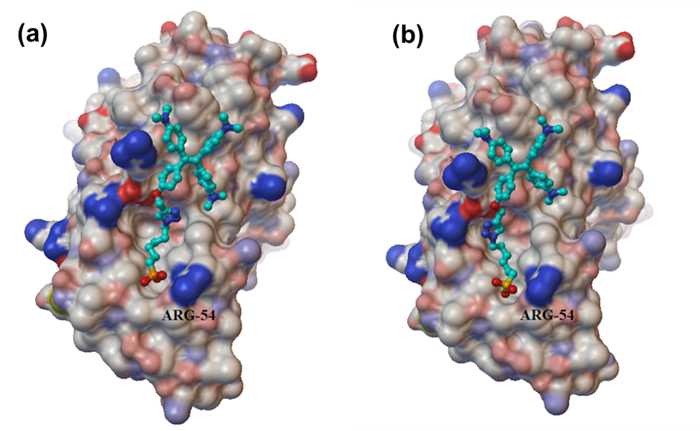


**Figure SIII**: Molecular docking of (a) TPE-P & (b) TPE-S in the active site of N-terminus domain of SARS-CoV-2 spike protein. The protein is shown in with molecular surface representation. The TPE-P & TPE-S molecules are shown in ball and stick model and the Arg54 amino acid residue location is shown on the protein.


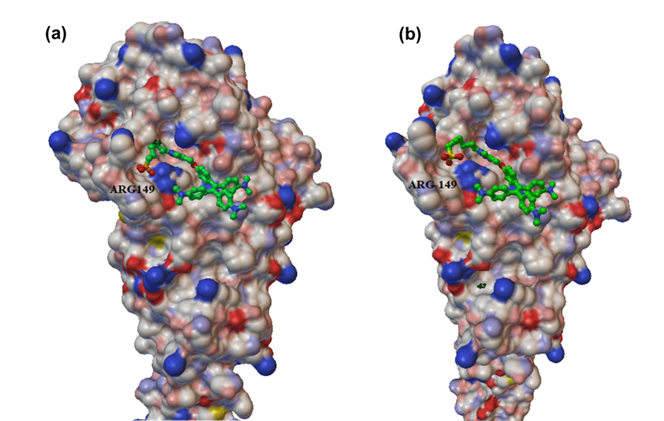


**Figure-SIV**: Molecular docking of (a) TPE-P & (b) TPE-S in the active site of H5N1 haemagglutinin protein. The protein is shown in with molecular surface representation. The TPE-P & TPE-S molecules are shown in ball and stick model and the Arg149 amino acid residue location is shown on the protein.


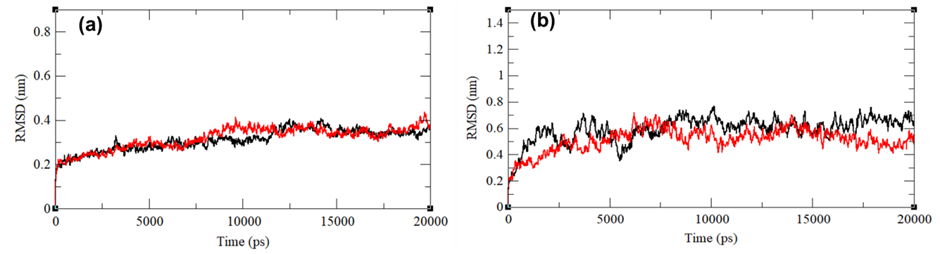


**Figure-SV**: (a) The RMSD plots (nm) ACE2 binding domain of SARS-CoV-2 spike protein in complexed with TPE-P molecule (black) and TPE-S molecule (red) during the 20 nano seconds of simulation. (b) The RMSD plots (nm) H5N1 haemagglutinin protein in complexed with TPE-P molecule (black) and TPE-S molecule (red) during the 20 nano seconds of simulation.
